# Genome-Wide Identification and Functional Characterization of GATA Transcription Factor Gene Family in *Alternaria alternata*

**DOI:** 10.3390/jof7121013

**Published:** 2021-11-26

**Authors:** Yanan Chen, Yingzi Cao, Yunpeng Gai, Haijie Ma, Zengrong Zhu, Kuang-Ren Chung, Hongye Li

**Affiliations:** 1 The Key Laboratory of Molecular Biology of Crop Pathogens and Insects of Ministry of Agriculture and Rural Affairs, The Key Laboratory of Biology of Crop Pathogens and Insects of Zhejiang Province, Institute of Biotechnology, Zhejiang University, Hangzhou 310058, China; yananchen@zju.edu.cn (Y.C.); 15077853913@163.com (Y.C.); gaiy@zju.edu.cn (Y.G.); haijie_ma@163.com (H.M.); zrzhu@zju.edu.cn (Z.Z.); 2 School of Agriculture and Food Sciences, Zhejiang Agriculture & Forestry University, Hangzhou 311300, China; 3 Hainan Institute, Zhejiang University, Sanya 572000, China; 4 Department of Plant Pathology, College of Agriculture and Natural Resources, National Chung-Hsing University, Taichung 40227, Taiwan; krchung@dragon.nchu.edu.tw

**Keywords:** *Alternaria alternata*, GATA transcription factor, systematic analysis, fungal development, pathogenicity, RNA-seq

## Abstract

In the present study, we identified six GATA transcription factors (AaAreA, AaAreB, AaLreA, AaLreB, AaNsdD, and AaSreA) and characterized their functions in response to environmental stress and virulence in the tangerine pathotype of *Alternaria alternata*. The targeted gene knockout of each of the GATA-coding genes decreased the growth to varying degrees. The mutation of *AaAreA*, *AaAreB*, *AaLreB*, or *AaNsdD* decreased the conidiation. All the GATA transcription factors were found to be required for tolerance to cumyl hydroperoxide and tert-butyl-hydroperoxide (oxidants) and Congo red (a cell-wall-destructing agent). Pathogenicity assays assessed on detached citrus leaves revealed that mutations of *AaAreA*, *AaLreA*, *AaLreB*, or *AaNsdD* significantly decreased the fungal virulence. A comparative transcriptome analysis between the ∆*AreA* mutant and the wild-type strain revealed that the inactivation of *AaAreA* led to alterations in the expression of genes involved in a number of biological processes, including oxidoreductase activity, amino acid metabolism, and secondary metabolite biogenesis. Taken together, our findings revealed that GATA-coding genes play diverse roles in response to environmental stress and are important regulators involved in fungal development, conidiation, ROS detoxification, as well as pathogenesis. This study, for the first time, systemically underlines the critical role of GATA transcription factors in response to environmental stress and virulence in *A. alternata*.

## 1. Introduction

Transcription factors (TFs) are a group of proteins that bind to specific DNA-regulatory sequences, thereby controlling the transfer of genetic information from DNA to mRNA. TFs are grouped into different families based on their DNA-binding domains [1]. The GATA TFs contain one or two highly conserved zinc finger DNA-binding domains, which can bind to the elements with consensus sequence (A/T)GATA(A/G) [2,3]. GATA TFs are broadly distributed in animals, plants, and fungi; however, their numbers and structures vary greatly within and between different kingdoms [3]. In animals, GATA TFs typically possess two adjacent homologous zinc fingers. Only the carboxyl-terminal finger is involved in DNA binding, whereas the amino-terminal finger interacting with other proteins is a determinant of specificity [4,5]. Animal GATA TFs have long been known to play important roles in the development, differentiation, and proliferation of cells [6,7]. In plants, most GATA TFs contain a single zinc finger domain, and only few harbors two zinc finger domains [8,9]. Plant GATA TFs have been demonstrated to play vital roles in light response, nitrogen metabolism, and phytohormone signaling [10,11]. In fungi, the majority of GATA TFs contain a single zinc finger domain [12]. Fungal GATA TFs have been shown to be involved in diverse functions, such as nitrogen metabolism, siderophore biosynthesis regulation, light regulation, mating-type switching, and chromatin rearrangements [13].

In contrast to animals and plants, fungi possess relatively fewer GATA TFs. However, few systematical studies have been carried out to determine the functions of GATA homologs in fungi, even though variations in the functions of GATA homologs have been speculated in different fungi. In *Aspergillus nidulans,* six GATA TFs (SreA, AreA, AreB, LreA-LreB, and NsdD) have been characterized. SreA acts as a repressor involved in the regulation of siderophore biosynthesis and iron transport [14]. AreA, which remodels the chromatin structure in the promoter of gene encoding niiA–niaD (nitrite and nitrate reductase), is a decisive regulator of nitrogen metabolism [15]. AreB has pleiotropic functions, negatively regulating nitrogen catabolism and positively functioning in growth, conidial germination, and asexual development [16,17]. In *A. nidulans*, LreA and LreB form a heterodimer, which, in turn, interacts with phytochrome FphA and the VeA regulator to form a large protein complex in the nucleus to sense red and blue light and regulate morphological and physiological differentiation [18]. NsdD plays a vital role in sexual reproduction [19]. Recently, an SWI/SNF chromatin-remodeling complex subunit snf5 has been found, for the first time, to contain a GATA domain in *Aspergillus oryzae* [20]. In the budding yeast *Saccharomyces cerevisiae*, four GATA TFs are involved in nitrogen metabolism. Among them, Gln3 and GAT1 are positive regulators, and Dal80p and GFZ3p are negative regulators [21]. In *Neurospora crassa*, WC1 and WC2 form a dimer to regulate genes involved in light-sensing [22,23]. Those studies indicate that the number and function of GATA TFs are highly diverse among different fungi. Little has been known about the GATA TFs in plant pathogenic fungus *Alternaria alternata*. Only SreA, acting as a siderophore repressor, has recently been characterized to be involved in the growth, hydrogen peroxide resistance, and cell wall integrity in *A. alternata* [24].

*A. alternata* is an economically important plant pathogen, which has been reported to cause diseases in over 100 plant species. Many *A. alternata* pathotypes are necrotrophic fungi, which have evolved to develop the ability to secrete host selective toxins (HST), which can kill host cells before invasion and colonize various economically important crops, such as Japanese pear, strawberry, tangerine, apple, tomato, rough lemon, and tobacco [25,26]. The tangerine pathotype of *A. alternata* is an important citrus pathogen, which causes Alternaria brown spot on tangerines, grapefruit, and their hybrids, resulting in yield losses [27]. This pathogen initiates its infection by producing an HST termed ACT (*A. citri* toxin), which is mainly synthesized by proteins encoded by multiple ACT genes located on the ACT biosynthesis gene cluster [26,27]. It has been well known that the genes involved in ACT biosynthesis are clustered in a small, conditionally dispensable chromosome [28,29]. In addition to ACT, the ability to produce cell-wall-degrading enzymes (CWDEs) and detoxify reactive oxygen species (ROS) is crucial for the successful penetration and colonization of *A. alternata* [30,31,32,33].

Previous studies have demonstrated that the AaSreA GATA TF is required to protect fungal cells from cytotoxicity caused by excess iron [24]. However, genetic dissection of GATA TFs in *A. alternata* has never been systematically studied. In this work, we identified six putative GATA-coding genes (*AaAreA*, *AaAreB*, *AaLreA*, *AaLreB*, *AaNsdD*, and *AaSreA*) in the tangerine pathotype of *A. alternata*. By creating the gene-knockout mutants, we have characterized the functions of these GATA TFs to be required for vegetative growth, conidiation, and stress responses. Significantly, we have demonstrated that, except AaAreB and AaSreA, all four *A. alternata* GATA (AaGATA) TFs are required for plant infection. A transcriptome analysis was also conducted to explore the regulatory role of AaAreA in the development of this important citrus pathogen. Combining the phenotypical characterization and gene expression of *AaGATA* mutants relative to the wild-type strain provides a unique opportunity to define the biological function of AaGATA TFs and expand our understanding of how *A. alternata* utilizes GATA TFs to deal with environmental stress and to attack its host plant. Understanding the mechanisms by which fungal pathogens cause diseases may help develop new fungicides to contain them in order to reduce yield loss and to ensure food security.

## 2. Materials and Methods

### 2.1. Fungal Strains and Culture Conditions

The wild-type Z7 strain of *A. alternata* (CGMCC3.18907), originally isolated from an infected tangerine ‘Ougan’ (*Citrus suavissima* Hort. Ex Tanaka) from Zhejiang Province, China [34,35,36], was used for transformation and mutagenesis. All fungal strains were grown on potato dextrose agar medium (PDA, 200 g potato, 20 g glucose, and 20 g agar per liter of purified water) and minimal medium (MM; 0.5 g KCl, 2 g NaNO_3_, 1 g KH_2_PO_4_, 0.5 g MgSO_4_·7H_2_O, 0.01 g FeSO_4_, 10 g sucrose, 200 mL trace elements, and 20 g agar per liter) at 26 °C to evaluate their growth and colony characteristics. Trace elements solution consists of 5 g of ZnSO_4_, 5 g of citric acid, 0.25 g of CuSO_4_·5H_2_O, and 1 g of (NH_4_)_2_Fe(SO_4_)_2_·6H_2_O per 100 mL. A regeneration medium (RMM) containing sucrose was used to recover fungal transformants. Potato dextrose broth (PDB) medium contains 200 g potato, 20 g glucose, and 1 L of purified water. Conidia were harvested from fungal cultures grown on V8 medium (200 mL V8 broth, 3 g CaCO_3_, and 20 g agar per liter) for 8 days by immersing, scraping with sterile water, and passing through three layers of sterile cheesecloth. Phenotypic assays were performed on PDA amended with a test compound. Fungal conidia and hyphae were examined with a Nikon microscope equipped with an LV100ND image system (Nikon, Tokyo, Japan).

### 2.2. Targeted Gene Disruption and Complementation

The *A. alternata* GATA transcription factors were identified using the *S. cerevisiae* and *A. nidulans* GATA homologs as a query to search the proteome of *A. alternata* by BLASTp. All deletion mutants were generated by a split-marker approach and protoplast transformation as previously described [32]. In brief, two DNA fragments fused with truncated hygromycin-resistance gene were amplified by fusion PCR and transformed directly into protoplasts prepared from the Z7 strain with CaCl_2_ and polyethylene glycol. Putative transformants were recovered from a regeneration medium (RMM) containing 100 mg/mL hygromycin. The resistant transformants were examined by PCR using the inner primers of AaGATA gene (AaGATA-F/AaGATA-R) and flanking primer of AaGATA gene pairing with a Hyg-specific primer (uup-F/nn-R). At least 10 independent transformants from each of the GATA TF-coding gene deletions were recovered and examined by PCR and phenotypical comparisons. Phenotypes of Δ*AaAreA* were restored by introducing and re-expressing a functional copy of *AaAreA* with its own promoter in pNEO1300 vector into Δ*AaAreA*. Complementation strains were selected on medium containing G418 and tested for growth and other phenotypes compared with wild-type and Δ*AaAreA*. All the primers used to generate fragments and for diagnostic PCR were included in Table S1.

### 2.3. Stress Adaptation Assays

For stress adaptation assays, all strains were grown on PDA medium supplemented with a metal ion stressor (1 mM CuSO_4_), an osmotic stressor (1 M NaCl or 1 M KCl), an oxidative agent (2 mM diethyl maleate (DEM), 1 mM cumyl hydroperoxide (CHP), 2 mM tert-butyl-hydroperoxide (t-BHP)), or a cell-wall-damaging agent (250 mg/mL Congo red). Each plate was inoculated with a 5-mm mycelial plug taken from the edge of a 5-day-old colony. Colony diameters were measured after culturing at 26 °C for 7 days. All assays were repeated twice, with three replicates per treatment.

### 2.4. Pathogenicity Assays

To assess fungal virulence, detached tangerine leaves ‘Hongjv’ were inoculated with 5-mm mycelial plugs taken from PDA, and the treated leaves were placed into a plastic box at 26 °C for 3 days. Each strain was tested on at least 12 leaves, and the pathogenicity experiments were repeated three times on the different days. Symptoms were recorded and the size of necrotic lesions quantified by ImageJ software (National Institutes of Health, Bethesda, MD, USA).

### 2.5. Transcriptome Analysis

Mycelial plugs of 3-day-old colonies of the *A. alternata* wild-type and Δ*AaAreA* strains were cultured in 150-mL PDB and incubated at 26 °C, 160 rpm for 36 h on a rotary shaker. For RNA extraction, mycelia were filtered, freeze-dried, and ground into a fine powder in liquid nitrogen. Total RNA was extracted using Axygen RNA purification kit (Axygen Scientific, St. Louis, MO, USA) according to the manufacturer’s instructions. RNA-seq was performed using three biological replicates of each sample. Complementary DNA (cDNA) libraries were constructed using Ultra RNA Library Prep kit (catalog no. E7530L; NEB, Ipswich, MA, USA). Sequencing was performed using Illumina HiSeq2000 sequencer platform (Illumina Inc., San Diego, CA, USA) to generate 150 bp paired-end reads. Adaptors and low-quality reads were removed using Trimmomatic v.0.36 [37]. Sequences were mapped to the *A. alternata* Z7 genome using Hisat2 v. 2.0.5 [38], and the number of reads mapped to each gene was counted by FeatureCounts v. 1.5.0-p3 [39]. Differential expression genes (DEGs) were analyzed using DESeq2 R package and defined by an adjusted *p*-value < 0.01 and an absolute value of log2 fold change (log2FC) greater than 2. Gene ontology (GO) and Kyoto Encyclopedia of Genes and Genomes (KEGG) pathway were performed using ClusterProfiler R package [40]. Gene clusters associated with secondary metabolites were predicted using antiSMASH 5.0. [41]. DEGs and secondary metabolite (SM) gene clusters were visualized by Circos v0.69 [42].

### 2.6. Gene Expression Analysis

The wild-type Z7 and the *AaGATA* mutant strains were cultured in PDB and incubated at 26 °C on a rotary shaker set at 160 rpm for 36 h. For RNA extraction, fungal mycelia were harvested by passing through cheesecloth, freeze-dried, and ground into fine powder in liquid nitrogen. Total RNA was extracted by Trizol and used for reverse transcription to produce cDNA using HiScript II Q RT SuperMix kit (Vazyme Biotech Co., Nanjing, China). Real-time quantitative PCR was performed using ChamQ SYBR qPCR master mix kit (Vazyme Biotech Co., Nanjing, China). The actin coding gene was used as a reference. Bio-Rad CFX96 real-time PCR detection system was used for qRT-PCR with an initial denaturation at 95 °C for 30 s, followed by 40 cycles of denaturation at 95 °C for 10 s and annealing and extension at 60 °C for 30 s. Each experiment was repeated three times using a comparative Ct method.

### 2.7. Statistical Analysis

All datasets and plotting were performed using IBM SPSS statistics 25 (IBM, Armonk, NY, USA) and Prism 9.0 (GraphPad Software Inc., San Diego, CA, USA). Significance of the treatments was determined by analysis of variance and treatment means separated by Duncan’s *t*-test (*p* ≤ 0.05).

## 3. Results

### 3.1. Identification of GATA Proteins in A. alternata

**Figure 1:**
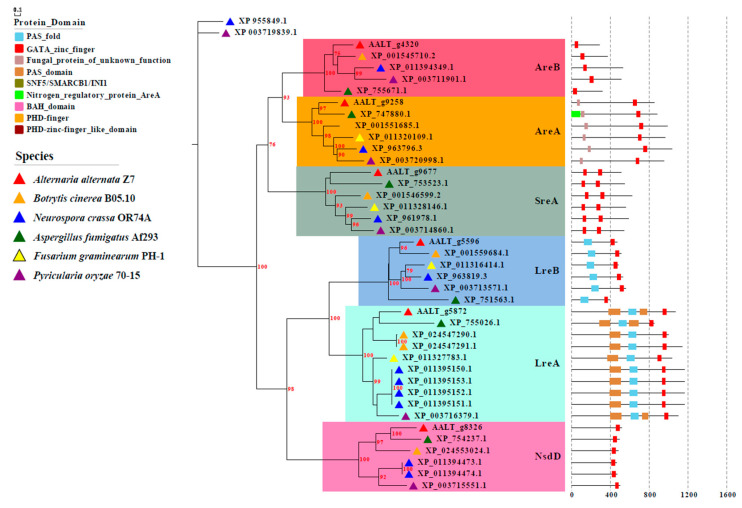
Phylogenetic analysis of AaGATA proteins. Amino acid sequences of GATA proteins were aligned with Clustal W, and MEGA7 was used to construct a neighbor-joining (NJ) tree, including bootstrap analysis with 1000 replicates. All positions containing gaps and missing data were eliminated. The domains of all proteins were identified by InterPro (http://www.ebi.ac.uk/interpro/, 17 November 2020). The evolutionary tree and protein domains were created by EvolView v2.0 (https://evolgenius.info/evolview-v2/, 20 November 2020).

Using *A. nidulans* GATA sequences as a query to carry out a genome-wide search for GATA proteins in the *A. alternata* genomic database (GenBank accession no. GCA_001572055.1; http://www.zjudata.com/alternaria/blast.php/, 11 August 2020) retrieved six homologous proteins named AaAreA, AaAreB, AaLreA, AaLreB, AaNsdD, and AaSreA. The protein domain analysis based on the InterPro database (https://www.ebi.ac.uk/interpro/, 12 August 2020) revealed that AaAreA (OWY57112), AaAreB (OWY51034), and AaNsdD (OWY45364) contain only one ZnF_GATA domain. AaSreA (OWY58354) contains two ZnF_GATA domains separated by 105 amino acid residues. AaLreB (OWY43847) contains a PAS domain in the N terminus and a ZnF_GATA domain in the C terminus. AaLreA (OWY44150) contains three PAS domains and a ZnF_GATA domain. The sequence alignment and phylogenetic analysis of the GATA protein homologs of different fungi showed that the domains of different GATA proteins are highly conserved among filamentous fungi. Amino sequences of AreB and LreB in *A. alternata* were most similar to those of *Botrytis cinerea* (Figure 1). To gain insight into the functions of AaGATA proteins, each of the six genes encoding respective AaGATA proteins was independently deleted using the homology recombination strategy. More than 10 fungal transformants of each gene were identified and grown on PDA amended with hygromycin. Successful deletion in the respective gene was examined by PCR with gene-specific primer pairs (Figure S1). For each gene deletion, at least three hygromycin-resistant transformants were tested for phenotypes, revealing that all the test transformants from the same gene deletion displayed similar deficiency phenotypes (Figure S2). Only one representative of each gene is shown.

### 3.2. AaGATA Proteins Are Required for Vegetative Growth and Conidiation

**Figure 2:**
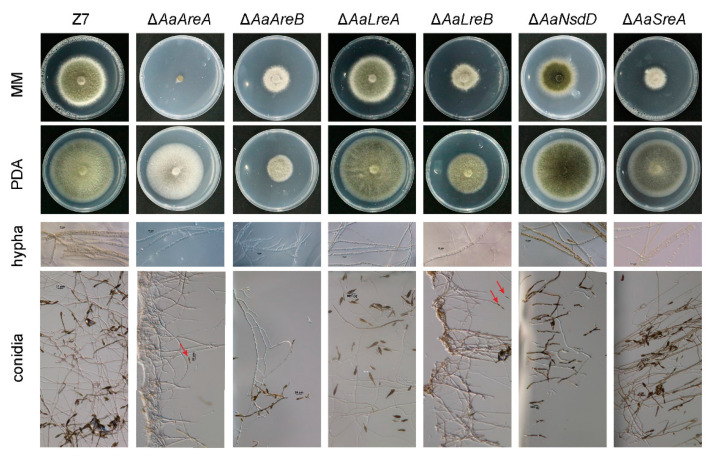
AaGATA transcription factors are involved in growth and development in *A. alternata*. Colony morphology was observed by incubating Z7 (wild-type), Δ*AaAreA*, Δ*AaAreB*, Δ*AaLreA*, Δ*AaLreB*, Δ*AaNsdD*, and Δ*AaSreA* on PDA or MM at 26 °C for 7 days and photographed. Hyphae, after being grown on PDA slab agars for 36 h, were examined microscopically. Bar = 10 μm. Conidial formation was observed after induction on V8 juice agar in the dark for 8 days. All tests were repeated at least twice with three replicates of each treatment.

**Figure 3:**
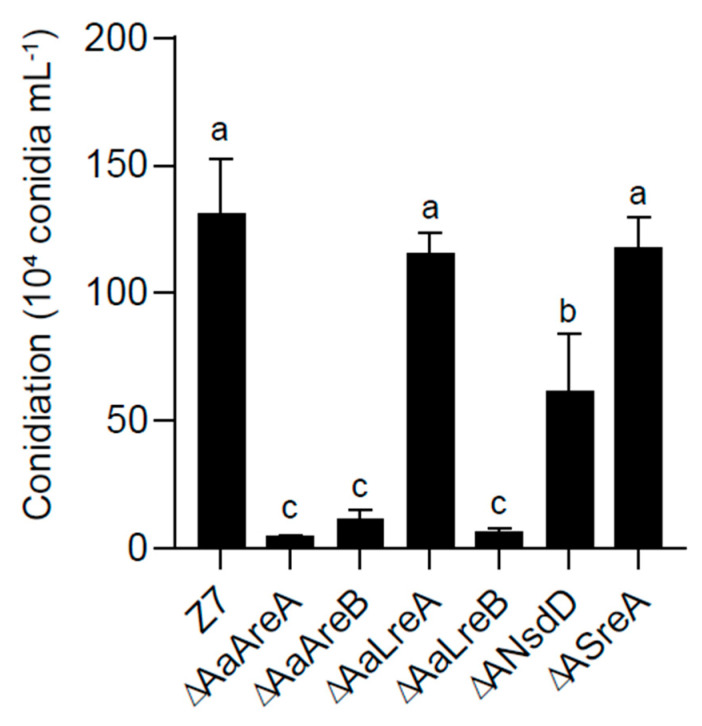
Quantitative analysis of conidia produced by *A. alternata* strains. The wild–type Z7, Δ*AaAreA*, Δ*AaAreB*, Δ*AaLreA*, Δ*AaLreB*, Δ*AaNsdD*, and Δ*AaSreA* strains were grown on V8 juice agar at 26 °C in the dark for 8 days. All tests were repeated at least twice with three replicates of each treatment. Same letters indicate non–significant difference estimated by Duncan’s test (*p* ≤ 0.05).

The colony morphology of the *AaGATA* mutants was significantly different from that of the wild-type Z7 strain grown on artificial medium. On PDA, Δ*AaLreA* displayed the wild-type radial growth. However, the radial growth of Δ*AaAreB*, Δ*AaLreB*, Δ*AaNsdD*, Δ*AaAreA*, and Δ*AaSreA* was reduced by 69%, 37%, 10%, 15%, and 22%, respectively, in relation to that of the wild-type strain. Δ*AaAreA* formed non-pigmented colonies with rare aerial hyphae on PDA. When grown on MM, the radial growth of all the mutants except Δ*AaLreA* was significantly reduced. Noticeably, Δ*AaAreA* barely grew on MM. The microscopic examination revealed that hyphal branching in Δ*AaAreB* was significantly increased compared to Z7 and other mutant strains (Figure 2). When grown on the V8 juice medium for 8 days, the conidial production of Δ*AaAreB*, Δ*AaLreB*, Δ*AaNsdD*, and Δ*AaAreA* was reduced by 91%, 95%, 53%, and 96%, respectively, compared with the wild-type strain (Figure 3). Δ*AaLreA* and Δ*AaSreA* slightly reduced the conidial production. The *AaAreA* complementation strain displayed wild-type growth, conidiation, and morphologies (Figure S2). Our results indicated that all the GATA proteins, except AaLreA, played important roles in the vegetative growth and conidia formation in *A. alternata*.

### 3.3. AaGATA Proteins Are Involved in Resistance to Multiple Stresses

**Figure 4:**
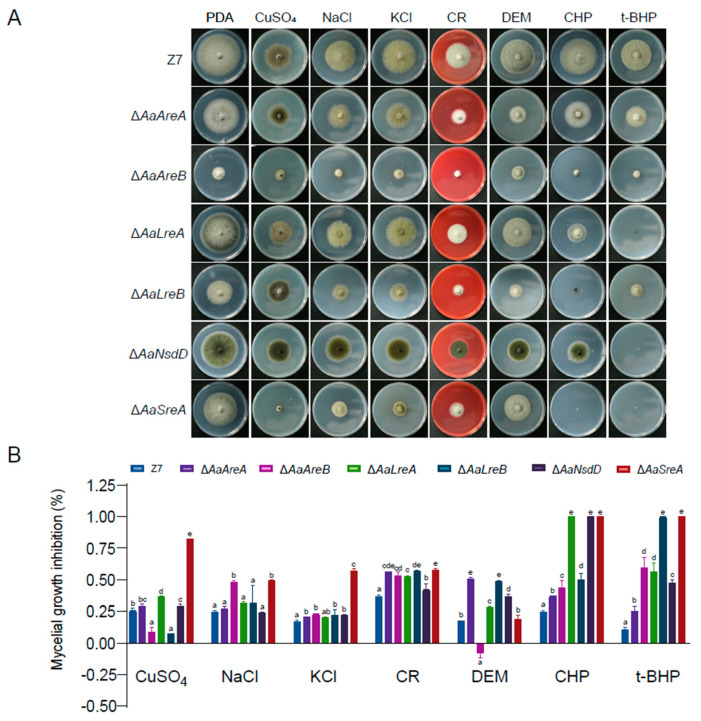
AaGATA transcription factors are required for stress resistance. (**A**) Mycelial plugs (5 mm) of Z7, Δ*AaAreA*, Δ*aaAreB*, Δ*aaLreA*, Δ*aaLreB*, Δ*aaNsdD*, or Δ*aaSreA* were inoculated on PDA or PDA containing 1 mM CuSO_4_, 1 M NaCl, 1 M KCl, 250 mg/mL Congo red, 2 mM diethyl maleate (DEM), 1 mM cumyl hydroperoxide (CHP), or 2 mM tert–butyl–hydroperoxide (t–BHP) for 7 days. (**B**) Growth inhibition rate was calculated. After 7–day inoculation, the diameter of colonies was measured to calculate the growth inhibition rate. Same letters in same stress item indicate non–significant difference estimated by Duncan’s test (*p* ≤ 0.05). All tests were repeated at least twice with three replicates of each treatment.

To assess the involvement of *AaGATA* genes in environmental adaptation, fungal mutants were assayed for resistance to stress conditions and compared to wild-type grown on PDA (Figure 4A). The percentages of growth inhibition compared to that of wild-type were calculated (Figure 4B). The results revealed that Δ*AaSreA* and, to a lesser extent, Δ*AaLreA* showed increased sensitivity to 1 mM CuSO_4_; however, Δ*AaAreB* and Δ*AaLreB* enhanced resistance to CuSO_4_ compared to Z7. The deletion of *AaAreA* or *AaNsdD* had little or no effect on the resistance to CuSO_4_. Only Δ*AaAreB* and Δ*AaSreA* increased the sensitivity to NaCl, and others displayed wild-type resistance. Only Δ*AaSreA* elevated the sensitivity to KCl, and other mutants had wild-type resistance. All the *AaGATA* mutants increased the sensitivity to Congo red (CR). Δ*AaAreA*, Δ*AaLreA*, Δ*AaLreB*, and Δ*AaNsdD* increased the sensitivity to diethyl maleate (DEM), whereas Δ*AaAreB* increased the tolerance and Δ*AaSreA* displayed wild-type resistance to DEM. All the *AaGATA* mutants increased the sensitivity to varying degrees to both 1 mM cumyl hydroperoxide (CHP) and 2 mM tert-butyl-hydroperoxide (t-BHP). The *AaAreA* complementation strain displayed wild-type sensitivity to all the test chemicals (Figure S2).

### 3.4. AaLreA, AaNsdD, and AaSreA Play Negative Roles in Gene Expression

**Figure 5:**
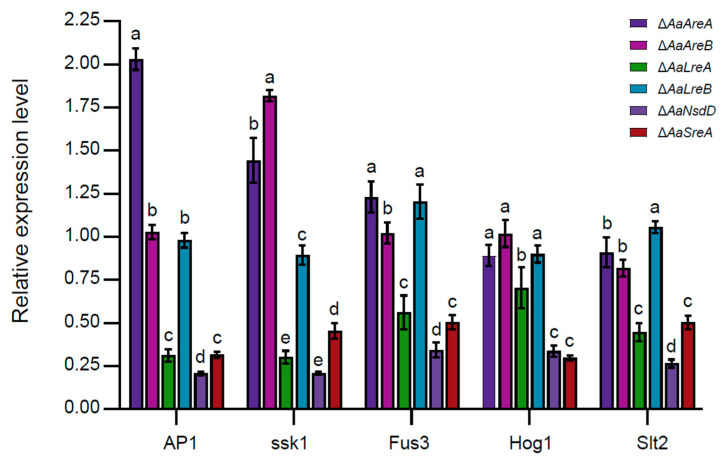
Quantitative real-time PCR (qRT-PCR) analysis. Expression of the genes encoding a redox transcription regulator AP1, a response regulator ssk1, as well as three mitogen-activated protein kinases, Fus3, Hog1, andSlt2, in the wild-type (WT) and six *AaGATA* mutants of *A. alternata*. The β-actin coding gene was used as a reference gene. The relative expression level of a gene in mutants was determined in relation to that of the wild-type using a comparative Ct method. Same letters in same stress item indicate non-significant difference estimated by Duncan’s test (*p* ≤ 0.05). All tests were repeated at least twice with three replicates of each treatment.

The quantitative RT-PCR analyses revealed that the deletion of *AaLreA*, *AaNsdD,* or *AaSreA* decreased the expression of *AP1* (encoding an oxidative stress regulator), *ssk1* (encoding a stress response regulator), and *Slt2* (encoding a MAP kinase involved in the maintenance of cell wall integrity). The deletion of *AaLreA*, *AaNsdD*, or *AaSreA* also decreased the expression of two MAP kinase-coding genes, *Fus3* and *Hog1*. The deletion of *AaAreA* increased the expression of *AP1* and, to a lesser extent, *ssk1* and *Fus3*, but had no or little effect on the expression of *Hog1* and *Slt2*. The deletion of *AaAreB* increased the expression of *ssk1* but had no effect on the expression of *AP1*, *Fus3*, *Hog1*, or *Slt2*. The deletion of *AaLreB* had no effect on the expression of the test genes (Figure 5).

### 3.5. AaGATA Proteins Are Required for Full Virulence

**Figure 6:**
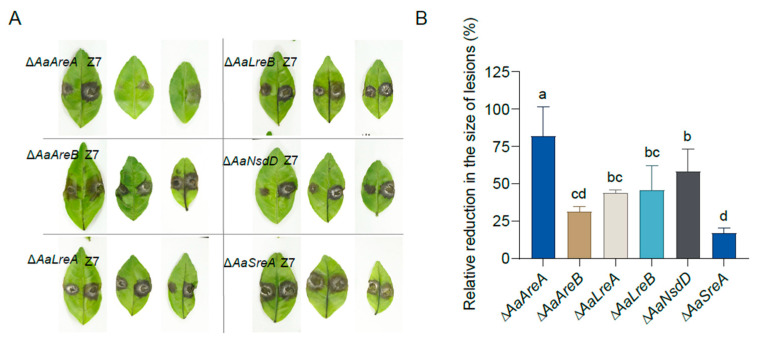
AaGATA transcription factors are required for *A. alternata* pathogenicity in citrus leaves. (**A**) Pathogenicity was assayed on detached ‘Hongjv’ leaves by placing a 5-mm agar plug covering fungal mycelium on the leaves. Inoculated leaves were incubated in a plastic box for lesion development. Photograph was taken 3 dpi. All tests were repeated at least twice with three replicates of each treatment. (**B**) The relative reduction (%) of necrotic lesions caused by the mutants compared to those induced by the wild-type strain Z7 was calculated by image J. Same letters in same stress item indicate non-significant difference estimated by Duncan’s test (*p* ≤ 0.05). All tests were repeated at least twice with three replicates of each treatment.

To determine the roles of AaGATA proteins in fungal virulence, detached ‘Hongjv’ leaves were inoculated with mycelial plugs taken from Z7 or various *AaGATA* mutants. The results indicated that Z7 caused typical brown necrosis around the inoculated point 3 days post-inoculation (dpi). The Δ*AaAreB* and Δ*AaSreA* strains induced necrotic lesions at a rate and magnitude comparable to those for the wild-type, whereas Δ*AaAreA*, Δ*AaLreA*, Δ*AaLreB*, and Δ*AaNsdD* induced significantly smaller lesions compared to those induced by the wild-type (Figure 6A). The quantitative analysis revealed that the sizes of the necrotic lesions induced by the Δ*AaAreA*, Δ*AaLreA*, Δ*AaLreB*, and Δ*AaNsdD* were reduced by 82%, 44%, 46%, and 59%, respectively, when compared to those induced by the wild-type (Figure 6B). The *AaAreA* complementation strain displayed wild-type virulence (Figure S2).

### 3.6. Transcriptome Analysis Defines the Global Regulatory Role of AaAreA in A. alternata

**Figure 7:**
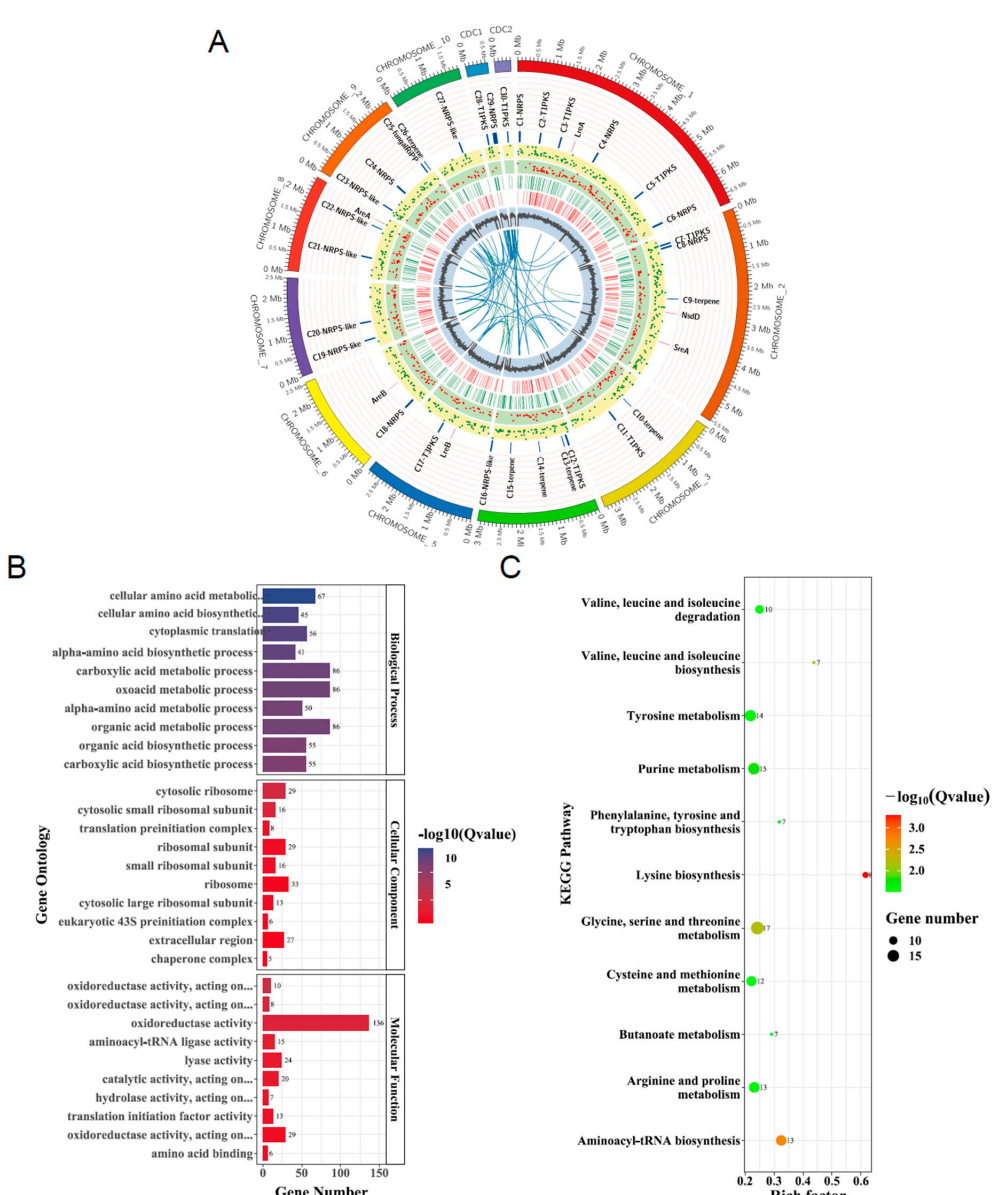
Transcriptome analysis of differentially expressed genes (DEGs) in Δ*AaAreA*. (**A**) Circos plot displaying the differences in gene expression and mRNA expression in Δ*AaAreA* mutant compared to Z7. Each circle from the periphery to the core represents: chromosomal location, secondary metabolite gene clusters, differentially expressed genes (DEGs), and GC content. The gene duplication is shown in the center. The conditionally dispensable chromosome (CDC) is also indicated. (**B**) Gene Ontology (GO) enrichment analysis of DEGs between Δ*AaAreA* and wild-type. (**C**) Scatter plots of KEGG pathway enrichment statistics based on statistical analysis of DEGs in Δ*AaAreA*.

As described above, AaAreA is not only involved in the fungal development but also plays an important role in the pathogenicity in *A. alternata*. To understand the regulatory landscape of AaAreA, we, therefore, compared the genome-wide gene expression of the wild-type and the Δ*AaAreA* strains by RNA-seq. The RNA-seq experiments were performed in biological triplicate for both strains. Overall, 1611 differentially expressed genes (DEGs), including 883 down-regulated and 728 up-regulated genes, were identified in Δ*AaAreA* compared to the wild-type Z7 (Table S2). A circos plots analysis showed the genomic location of the genes differentially expressed in the Δ*AaAreA* mutant, as well as the position and expression level of genes involved in secondary metabolite biosynthesis (Figure 7A). The transcriptome analysis revealed that the expression of four cutinase-coding genes, Aacut1 (AALT_g1915), Aacut5 (AALT_g1519), Aacut3 (AALT_g9295), and Aacut4 (AALT_g380), were significantly decreased in Δ*AaAreA* (Table S2). Comparative gene ontology (GO) and KEGG pathway analyses were performed to explore the function and regulatory role of DEGs in Δ*AaAreA*. After assigning these DEGs to different categories, the results revealed that most DEGs were significantly enriched in “carboxylic acid metabolic process”, “oxoacid metabolic process”, “organic acid metabolic process”, “cellular amino acid metabolic process”, “ribosome synthesis”, and “oxidoreductase activity” (Figure 7B). The KEGG enrichment analysis revealed that AaAreA is probably involved in the gene expression regulation of several critical metabolic pathways, including metabolism of tyrosine, purine, glycine, serine, threonine, cysteine, butanoate, methionine, arginine, proline, and biosynthesis of lysine, phenylalanine, tyrosine, tryptophan, valine, leucine, and isoleucine. In addition, some DEGs were enriched in aminoacyl-tRNA biosynthesis (Figure 7C).

**Figure 8:**
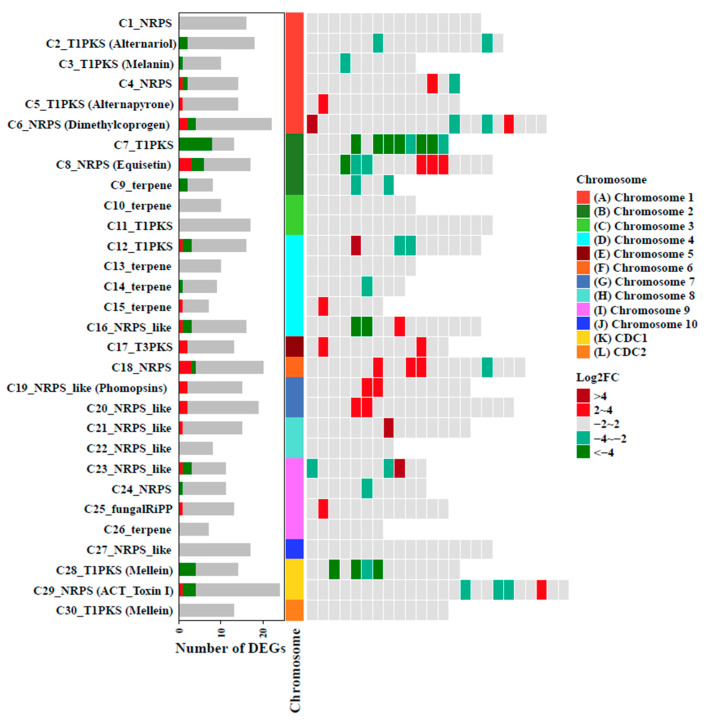
Differential expression of gene clusters associated with the secondary metabolite biosynthesis between the Δ*AaAreA* mutant and the wild-type Z7. Abbreviations: pks, polyketide synthase; nrps, non-ribosomal peptide synthetase; T1, type 1; T3, type 3; terpene, terpene synthetase.

To explore the relationships between the secondary metabolites and pathogenicity mediated by AaAreA, the transcriptional regulation of biosynthetic gene clusters in *A. alternata* were predicted by antiSMASH 5.0. The results revealed that AaAreA regulates a broad range of gene clusters involved in the biosynthesis of many secondary metabolites. Significantly, the expression of many genes in cluster 5 (T1PKS, Alternapyrone), cluster 15 (terpene), cluster 17 (T3PKS), cluster 18 (NRPS), cluster 19 (Phomopsins), and cluster 20 (NRPS) were highly upregulated in Δ*AaAreA*, suggesting that AaAreA is a negative regulator for the biosynthesis of secondary metabolites. However, the expression of many genes in cluster 2 (Alternariol), cluster 7 (T1PKS), cluster 28 (T1PKS, Mellein), and cluster 29 (ACT toxin) were strongly downregulated in Δ*AaAreA*, indicating that AaAreA regulates the biosynthesis of Alternariol and ACT toxin in a complex manner (Figure 8). The expression of six genes located in the ACT toxin gene cluster was validated further by qRT-PCR. The transcript levels of genes encoding ACTT3 (enoyl-CoA hydratases), ACTTR [Zn (II)2Cys6 transcription factor], and ACTTS3 (polyketide synthase) were decreased in Δ*AaAreA*, consistent with RNA-Seq (Figure S3).

## 4. Discussion

Alternaria brown spot is one of the most destructive diseases of the citrus industry. So far, the most effective control measure is still chemical control, and the disadvantages of fungicides on the environment and food safety are obvious. Therefore, there is an urgent need to develop new ways of disease control to make up for the existing shortcomings. Understanding the molecular mechanisms underlying the pathogenicity and identifying the key genes determining the pathogenicity would provide the targets for the development of a more specific fungicide against this pathogen. Transcription factors are sequence-specific DNA-binding proteins that bind to the promoters of specific genes and regulate their expression, thus conferring transcription factors with a wide range of functions in all living things. Only a few TFs have been characterized in *A. alternata*. Studies on *A. brassicicola* have identified and characterized a number of TFs and their roles in fungal virulence [43,44]. Understanding the functions of TFs in phytopathogenic fungi may lead to the identification of novel virulence factors and improve our ability to deal with fungal pathogens [45].

GATA TFs containing conserved zinc finger DNA-binding domains are ubiquitous in eukaryotes. To date, GATA proteins have only been systematically studied in a few fungal species, even though the number and biological function of GATA homologous proteins vary widely among fungal species [46,47,48,49,50,51]. In the present study, we have identified and functionally characterized six GATA transcription factor family genes (*AaAreA*, *AaAreB*, *AaLreA*, *AaLreB*, *AaNsdD*, and *AaSreA*) in the tangerine pathotype of *A. alternata*. As shown in Table S3, AaGATA TFs play diverse roles, either as a negative or positive regulator, in growth development, response to copper, salts, CR (a cell-wall-disrupting agent), DEM (a glutathione depletion agent), CHP and BHP (both are oxidants), and fungal virulence in *A. alternata*. All GATA-coding genes with the exception of *AaLreA* were found to contribute to the fungal vegetative growth and resistance to various stresses. *AaAreA*, *AaAreB*, *AaLreB*, and *AaNsdD*, but not *AaLreA* and *AaSreA*, play a role in conidia production. *AaAreA*, *AaLreA*, *AaLreB*, and *AaNsdD* also contributed to fungal virulence. The deletion of *AaAreA* led to expression changes in global genes, which function in many biological processes, including oxidoreductase activity, amino acid metabolism, and secondary metabolite biogenesis. This work represents the first comprehensive study on the functions of GATA transcription factors in *A. alternata*.

AreA and AreB are two GATA factors implicated in the regulation of nitrogen metabolism [52,53]. The *A. alternata* AaAreA and AaAreB are homologous to the *M. oryzae* NUT1 and ASD4 and the *A. nidulans* AreA and AreB, respectively. Our study demonstrated that AaAreA and AaAreB positively regulate the vegetative growth and conidiation in *A. alternata*, consistent with the findings in other fungi [16,54,55]. Compared to the wild-type, Δ*AaAreB* reduced growth on PDA and MM with different nitrogen sources at similar rates, indicating that AaAreA but not AaAreB plays a critical role in nitrogen metabolism. In *N. crassa*, WC-1 (LreA homolog) and WC-2 (LreB homolog), forming heterodimeric complexes to activate the transcription of frequency (frq), are two central components of the blue-light-sensing system [56,57]. In *A. nidulans*, the conidiospore production, independent of light, is slightly increased in the Δ*LreA* and Δ*LreB* strains [18]. In this study, we have shown that the deletion of *AaLreA* had no obvious effect on the conidiation. In contrast, the deletion of *AaLreB* showed a significant reduction in vegetative growth and conidiation, indicating that AaLreB plays a determinant role in the growth and development in *A. alternata*. The results also suggested that AaLreB plays a key role in light sensing. NsdD has been identified as an activator of sexual development and a repressor of asexual development in *A. nidulans* and *B. cinerea* [58,59]. We also found that AaNsdD is required for conidiation but not vegetative growth. Contradictory to AaNsdD, AaSreA is required for growth but not conidiation, which is consistent with the previous reports [24]. The results indicate that *A. alternata* utilizes multiple GATA TFs to coordinate vegetative growth and conidia biogenesis.

Some fungal GATA TFs have also been demonstrated to play important roles in response to abiotic stresses. Fungi have evolved sophisticated machinery to regulate Cu homeostasis to survive under host-imposed toxicity, and the maintenance of Cu homeostasis could be critical for fungal survival and pathogenesis [60,61]. In the human pathogen *Cryptococcus neoformans*, the GATA protein Cir1, a SreA homolog, controls copper uptake and homeostasis by regulating the expression of the copper exporting ATPase Ccc2 [49]. In this present study, we have shown that AaAreB and AaLreB negatively regulate cellular resistance to copper, and AaSreA positively regulates copper resistance. The results indicate the involvement of different GATA TFs in maintaining copper homeostasis to avoid copper toxicity in *A. alternata*. Moreover, AaSreA is also involved in the cellular resistance to salts because Δ*AaSreA* increased the sensitivity to NaCl and KCl. Previous studies have demonstrated that *A. alternata* could resist high concentrations of salts, mainly via the ssk1 and its downstream Hog1 MAPK-mediated pathway [62,63]. The inactivation of *AaSreA* downregulated the expression of the *Hog1* transcript (Figure 5), indicating that AaSreA regulates salt tolerance, likely via the Hog1-MAPK pathway. The ability to detoxify host-generated ROS is critical to the pathogenicity of many plant pathogens. Several fungal GATA proteins, such as LTF1, sfh1, and Csm1, have been demonstrated to be required to cope with oxidative stress and to maintain ROS homoeostasis [59,64,65]. With this in mind, we evaluated the response of the *AaGATA* mutants to various oxidants. Surprisingly, all the *AaGATA* mutants increased sensitivity to BHP and CHP. Previous studies have shown that *A. alternata* employs effective mechanisms to deal with the toxicity of ROS, and the ability to detoxify ROS is required for fungal invasion in the host plant. Several key regulators, including AaHog1, Aassk1, and AaAP1, have been shown to be required for ROS resistance and full virulence on citrus [33]. The quantitative RT-PCR analysis revealed that the deletion of *AaLreA*, *AaNsdD*, or *AaSreA* reduced the expression of *ssk1*, *AP1*, and *Hog1* (Figure 5), all implicated in ROS resistance. The results, at least in part, may explain why Δ*AaNsdD* and Δ*AaSreA* were more sensitive to ROS-producing compounds.

Many plant pathogenic fungi produce a wide variety of secondary metabolites with unique and complex structures, and many secondary metabolites are required for fungal pathogenicity [66]. It has been well known that the biosynthesis of secondary metabolites in phytopathogenic fungi is strongly affected by nitrogen availability. For instance, the biosynthesis of penicillin in *Penicillium chrysogenum*, trichothecenes and fusarielin H in *Fusarium graminearum*, and cephalosporin in *Acremonium chrysogenum* have been documented to be regulated by nitrogen [67,68,69,70]. AreA is a major nitrogen metabolism regulator that mediates the de-repression of the genes involved in the utilization of alternative nitrogen sources in the absence of preferred nitrogen sources [71]. Our results revealed that AaAreA and AaAreB play different roles in nitrogen regulation as the deletion of either gene led to different degrees of growth reduction on the medium with different nitrogen sources (PDA vs. MM).

The transcriptome analysis was performed to uncover the regulatory role of AaAreA in *A. alternata*. Of 1611 differently expressed genes identified, many are associated with metabolisms, particularly those related to amino acid metabolism. This finding may explain why the growth impairment of Δ*AaAreA* on MM is so dramatic. In addition to amino acid metabolism, our transcriptome data showed that AaAreA could affect the expression of the genes involved in the biosynthesis of multiple secondary metabolites. The results are consistent with the notion that AreA is a global transcription factor required for secondary metabolism in different fungi [72]. In total, 22 secondary metabolite (SM) gene clusters presumably regulated by AaAreA were identified. Many genes in cluster 2 (alternariol), cluster 28 (mellein), and cluster 29 (ACT toxin) were significantly down-regulated in Δ*AaAreA*. Several genes in cluster 17 (T3PKS) and cluster 18 were strongly up-regulated in Δ*AaAreA*. The transcriptome data also showed that AaAreA could affect the expression of the genes involved in the oxidation-reduction process, which may explain why that Δ*AaAreA* was sensitive to ROS-producing compounds. The deletion of *AaAreA* yielded fungal mutants that induced fewer lesions than the wild-type strain. The reduction in virulence may be partly due to the reduction in ACT biosynthesis and cutinase activity because the expression of several genes involved in the biosynthesis of ACT and the activity of cutinase were decreased in Δ*AaAreA.* The impairment of the expression of ACT biosynthetic genes and cutinase-encoding genes may contribute to the severe virulence defect seen in Δ*AaAreA.*

## 5. Conclusions

It the present study, we have functionally characterized six AaGATA proteins (AaAreA, AaAreB, AaLreA, AaLreB, AaNsdD, and AaSreA) in the tangerine pathotype of *A. alternata* and have found that AaGATA TFs play diverse roles in growth, conidiation, as well as stress tolerance and virulence. Despite their divergences in growth and development, all six GADT TFs are required for resistance to Congo red, cumyl hydroperoxide, and tert-butyl-hydroperoxide, suggesting an important role of GATA TFs in resistance to environmental stress. Such findings have never been reported to be associated with fungal GATA TFs. This study represents the first systemic investigation to define specific functions associated with each of the AaGATA proteins in *A. alternata*. Our results provide initial insights for further elucidating the precise molecular mechanism by which the AaGATA proteins affect fungal development, amino acid metabolism, secondary metabolites, and fungal virulence.

## Data Availability

The data presented in this study have been deposited at NCBI under BioProject accession PRJNA769496.

## Supplementary Materials

The following are available online at https://www.mdpi.com/article/10.3390/jof7121013/s1, Figure S1: Construction and confirmation of *AaGATA* disrupted mutants, Figure S2: Phenotypic confirmation of *AaGATA* mutants, Figure S3: Relative expression of six randomly selected genes in the ACT toxin gene cluster in Δ*AaAreA*, Table S1: Primers used in this study, Table S2: Differentially expressed genes (DEGs) in the *Alternaria alternata AaAreA* deficiency mutant, Table S3: Phenotypic summary of six *AaGATA* mutants.
