# Supplementary material for: Genome-Wide Identification and Functional Characterization of GATA Transcription Factor Gene Family in Alternaria alternata
Source: J Fungi (Basel). 2021 Nov 26;7(12):1013. doi: 10.3390/jof7121013 (PMC8706292; doi:10.3390/jof7121013)
Supplement: Supplementary file 1 [file jof-07-01013-s001.zip › supplementary figure.pdf]

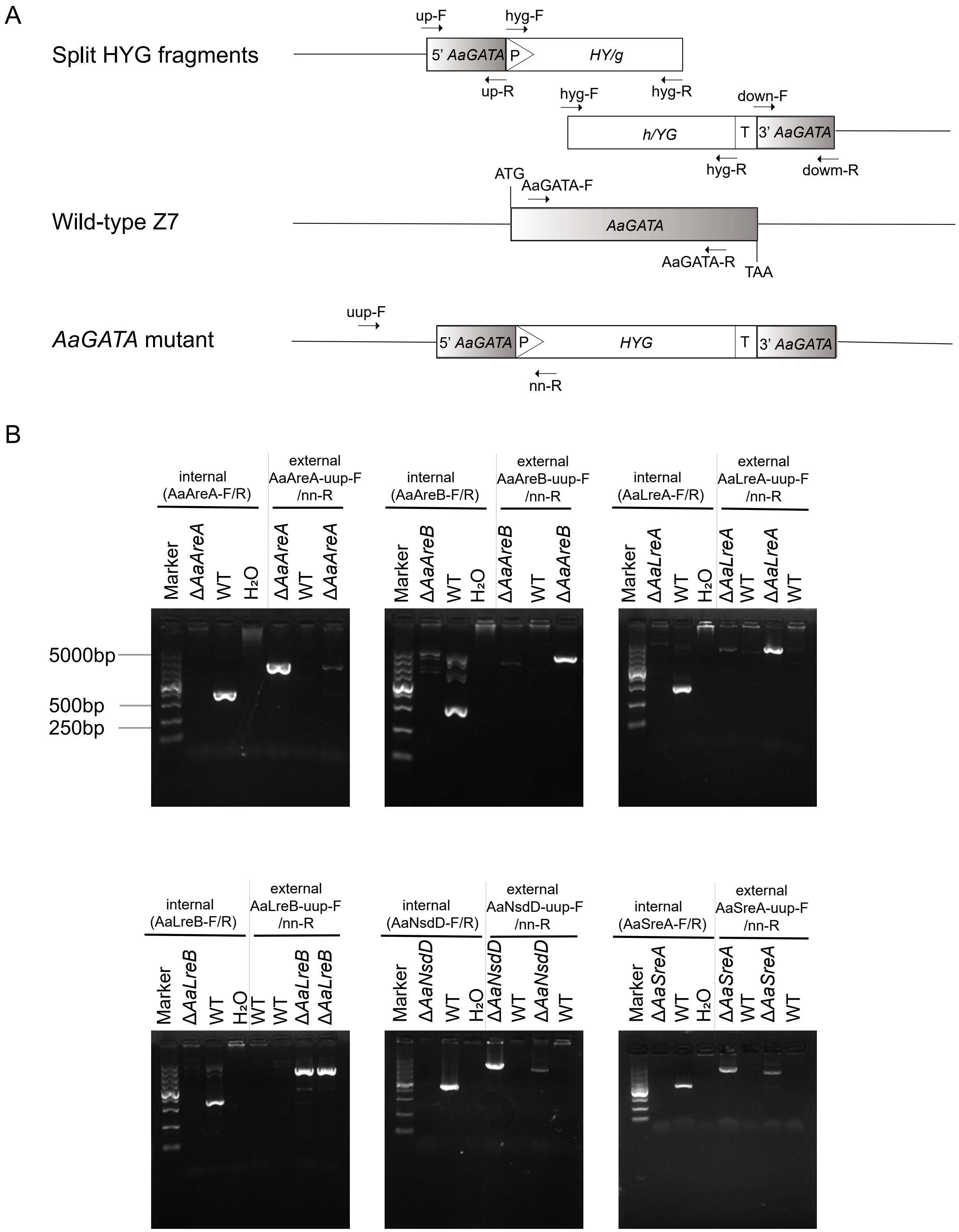


Figure S1. **Construction and confirmation of *AaGATA* disrupted mutants.** (a) Schematic depiction of the gene replacement strategy for *AaGATA*. All primers used in this study were list in Table S1. (b) PCR verification of the positive transformants with the primers indicated.

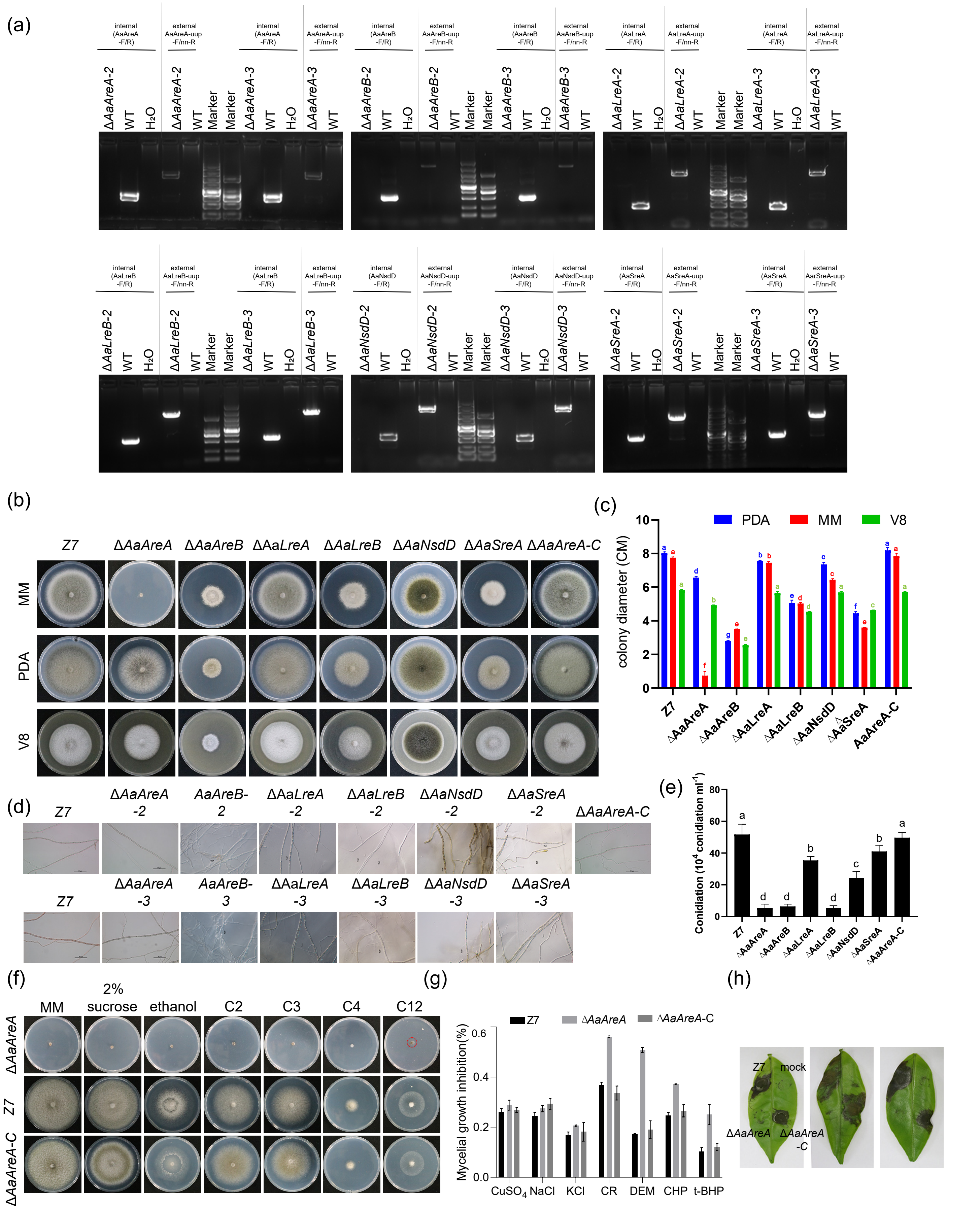


**Figure S2 Phenotypic confirmation of *AaGATA* mutants.** (a) PCR verification of two hygromycin-resistant transformants of each gene. (b) Three transformants of each *AaGATA* gene deletion mutant and Δ*AaAreA*-C were incubated on PDA, MM or V8 at 26°C for 7 days and colony diameters were measured. Only one representative transformant from each gene deletion was photographed for the colony morphology. (c) Quantification of colony diameter. Error bars represent standard deviations from three hygromycin-resistant transformants. (d) Hyphae of two hygromycin-resistant transformants of each gene were examined microscopically. (e) Quantitative analysis of conidia produced by *AaGATA* mutants. Error bars represent standard deviations from three hygromycin-resistant transformants. Different letters indicate statistical significance according to the one-way ANOVA test ($p < 0.05$). (f) Confirmation of Δ*AaAreA*-C complementation strain. Radial growth of the wild-type Z7, deletion mutant Δ*AaAreA* and complemented strain Δ*AaAreA*-C on minimal medium (MM) and modified MM supplemented with long-chain fatty acids as the sole carbon source. (g) Relative growth rate of Δ*AaAreA*, Z7, and Δ*AaAreA*-C. (h) Inoculation of Δ*AaAreA*, Z7, and Δ*AaAreA*-C by placing mycelial plugs on detached 'Hongjv' leaves. Blank agar plug was used as the mock.

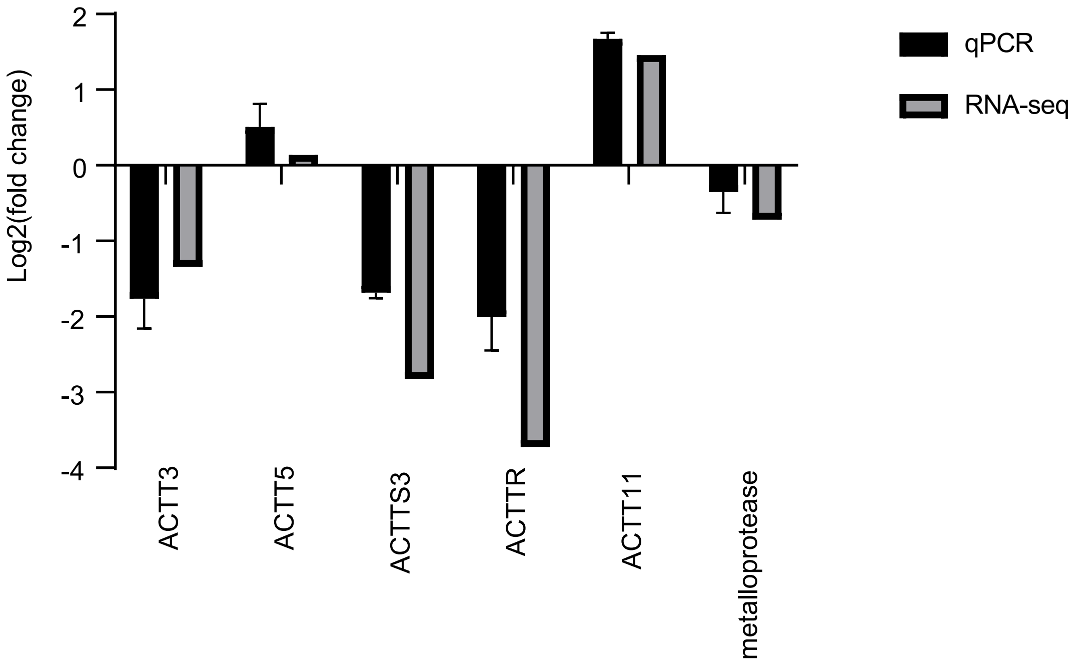


**Figure S3. Relative expression of six randomly selected genes in the ACT toxin gene cluster in Δ*AaAreA*.** The relative transcript levels of slected genes were analysed by RNA-Seq ($p$ < 0.05, log2FoldChange > 1). The β-actin coding gene was used as the reference gene. The relative expression level of a gene in Δ*AaAreA* was determined using a comparative Ct method in relation to that of the wild type.
